# Lemierre's syndrome: Pediatric clinical case presentation

**DOI:** 10.1016/j.radcr.2025.03.054

**Published:** 2025-04-12

**Authors:** Jennifer Richardson Maturana, Sara Gomez Milanés, Luz Angela Moreno Gómez, Liliana Henao Gomez

**Affiliations:** a Fundación Hospital Pediátrico La Misericordia, HOMI, Universidad Nacional de Colombia, Colombia; b Universidad Sanitas, Bogotá, Colombia; c Fundación Hospital Pediatrico La Misericordia, HOMI, Colombia

**Keywords:** Lemierre, Lemierre's syndrome, Fusobacterium necrophorum, Suppurative thrombophlebitis

## Abstract

Lemierre Syndrome (LS) is a rare infectious condition characterized by septic thrombosis of the internal jugular vein and septic embolization, mainly caused by Fusobacterium necrophorum. Diagnostic Imaging plays a key role in the early diagnosis and follow-up of the syndrome. This article reviews the available literature on radiology applied in LS and analyzes a pediatric clinical case that highlights the importance of computed tomography (CT) and magnetic resonance imaging (MRI) for identifying retropharyngeal abscesses and jugular thrombosis. The case involves a 2-month-old child diagnosed with LS complicated by mediastinitis, cervical empyema, and septic thrombosis of the internal jugular vein as complication.

## Introduction

Lemierre syndrome, also known as the “forgotten disease,” is a severe bacterial infection characterized by septic thrombosis of the internal jugular vein. Although it is a rare condition, it has gained attention in recent years due to a slight increase in its incidence and the severity of associated complications. This pathology was first described in 1936 by André Lemierre, who identified it as a condition typically affecting young adults, preceded by an acute bacterial oropharyngeal infection, usually caused by *Fusobacterium necrophorum* [1].

The clinical course of Lemierre syndrome usually begins with an upper respiratory tract infection, presenting typical symptoms such as fever, sore throat, and cervical lymphadenopathy [2]. In most cases, the primary infection affects the tonsils, pharynx, or middle ear. Without adequate treatment, the infection can spread to the blood vessels, resulting in thrombosis of the internal jugular vein, which can embolize to distant sites, primarily affecting the lungs [3].

## Case presentation

The patient, a 2-month-old infant, was admitted to the Pediatric Intensive Care Unit (PICU), presenting with acute respiratory failure due to a massive retropharyngeal abscess and septic thrombosis of the left internal jugular vein. The clinical course was significantly complicated by the onset of septic shock.

Initially, the patient had been hospitalized, for acute bronchiolitis associated with SARS-CoV-2, which rapidly led to severe complications. During the hospital stay, the patient developed a rapidly growing left cervical mass that extended into the mediastinum. This expansion critically compromised the airways, resulting in acute respiratory failure requiring invasive mechanical ventilation. Imaging studies confirmed the presence of an extensive retropharyngeal abscess and septic thrombosis of the left internal jugular vein, characteristic manifestations of Lemierre syndrome, a rare and severe condition in children.

### Medical history

The patient is the second child of a 28-year-old mother. He was born vaginally at 39 weeks of gestation, weighing 3830 grams, without perinatal complications or the need for admission to the Neonatal Intensive Care Unit. Prior to his hospitalization for bronchiolitis, he had contracted COVID-19 at 28 days of life. His vaccination schedule was up to date for his age, including 2 doses of the COVID-19 vaccine. No relevant family history was reported.

### Current illness

The patient was admitted to the PICU after his respiratory status significantly worsened. The left cervical mass, initially detected during his hospitalization, progressed rapidly, extending into the mediastinum and compressing the upper airways. This compression led to respiratory failure, requiring support with mechanical ventilation. Radiological studies confirmed the extension of the retropharyngeal abscess from the first cervical vertebra (C1) to the sixth thoracic vertebra (T6), with a maximum diameter of 103 × 11 mm, involving the deep spaces of the neck and mediastinum. This condition was further complicated by septic thrombosis of the left internal jugular vein, a hallmark finding of Lemierre syndrome.

### Clinical and imaging findings

Upon admission, the patient weighed 5.5 kg and had vital signs showing a heart rate of 122 beats per minute, respiratory rate of 28 breaths per minute, oxygen saturation of 95%, blood pressure of 95/56 mmHg, and body temperature of 36.5°C. A palpable left cervical mass was noted.

On respiratory examination, the child exhibited laryngeal stridor due to airway obstruction, prompting the use of high-flow nasal cannula. With steroid treatment and noninvasive ventilatory support, his respiratory pattern improved. Abdominal examination showed no signs of peritoneal irritation, and he tolerated enteral feeding well. Neurologically, the patient responded to tactile stimulation but not to verbal cues and exhibited symmetrical limb movements.

We prefer to perform contrast-enhanced magnetic resonance imaging of the neck and chest, without sedation, instead of computed tomography, due to its high spatial resolution in characterizing the extent of mediastinal and cervical involvement, and to avoid radiation in pediatric patients.

**Fig. 1:**
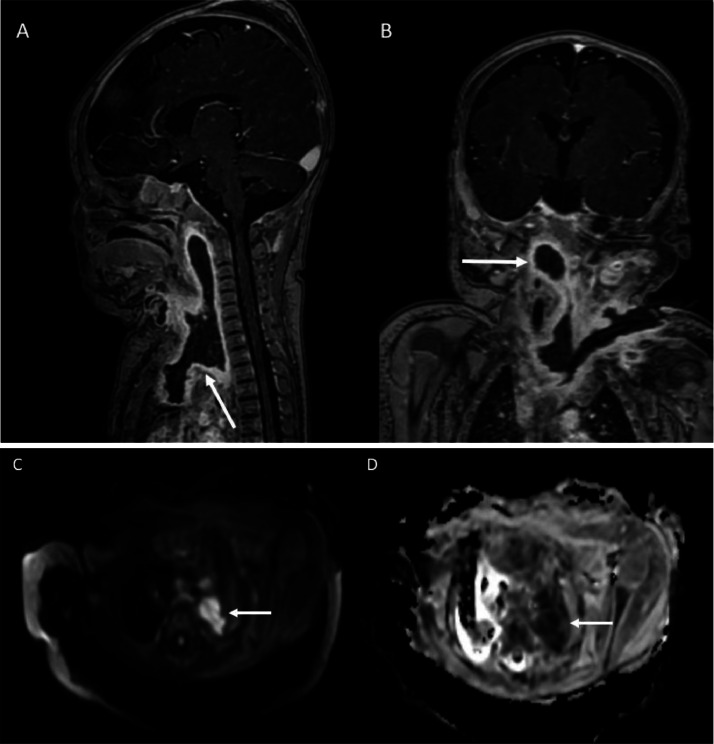
T1-weighted magnetic resonance images with intravenous contrast. Sagittal (A) and coronal views (B), demonstrate a large hypointense lesion with peripheral enhancement extending from the prevertebral space at the level of C1 to T6, to the supraclavicular regions, compromising the deep spaces of the neck: carotid, parapharyngeal, submandibular, retropharyngeal and perivertebral spaces as well as the mediastinal compartments. Note abscess and empyema formation in the pulmonary apices, which presents diffusion restriction in the DWI images (C) and its respective representation in the ADC map (D).

**Fig. 2:**
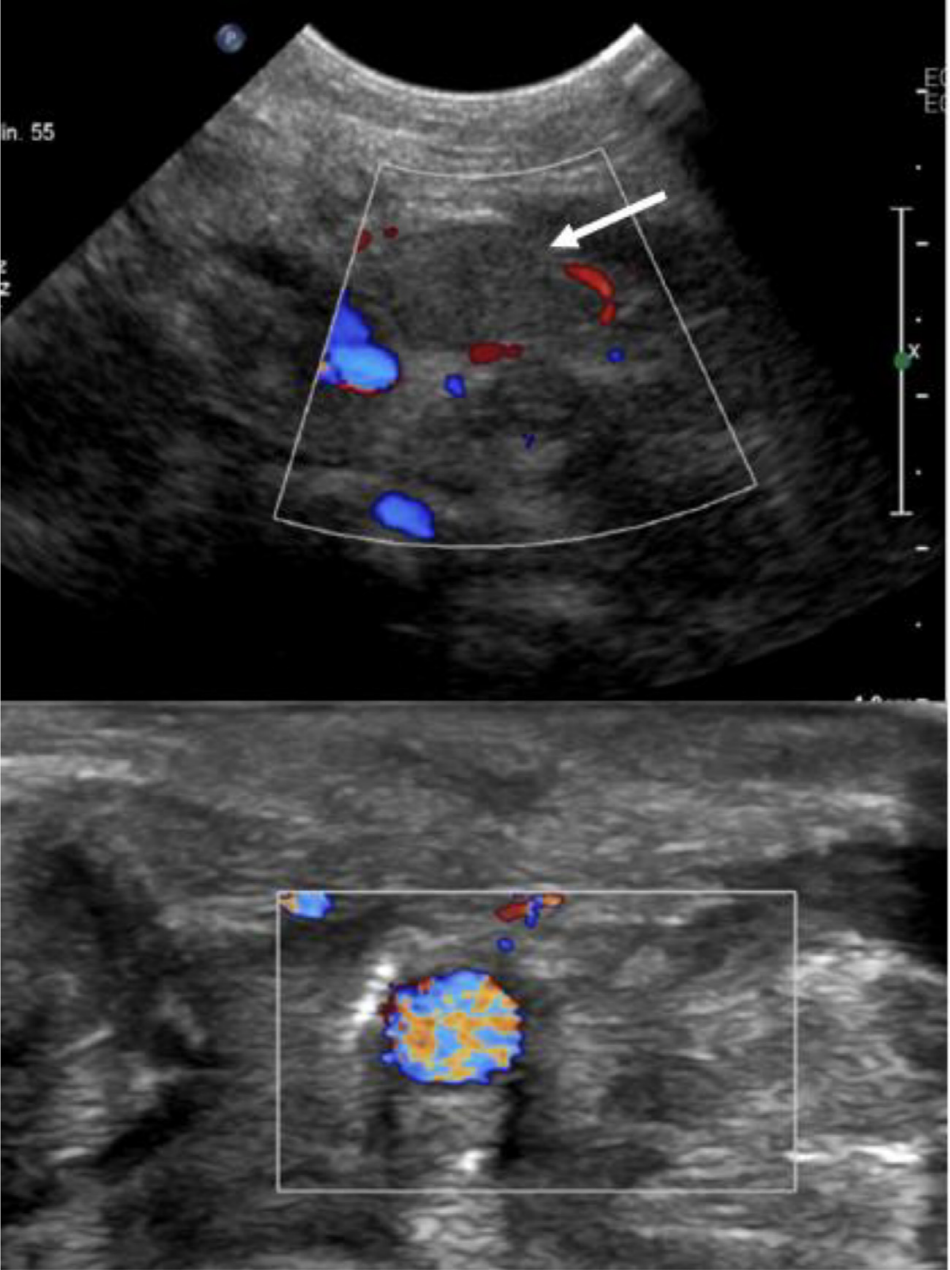
Doppler ultrasound of the neck vessels, shows high density material obstructing and distending the left jugular vein consistent with chronic thrombosis.

Imaging studies included a neck and chest magnetic resonance imaging (MRI), which revealed an organized retropharyngeal abscess extending from C1 to T6, involving the supraclavicular, parapharyngeal, submandibular, and mediastinal regions. This abscess was accompanied by septic thrombosis of the left internal jugular vein (Fig. 1). MRI confirmed the extent of the abscess and the presence of jugular thrombosis. In addition, local inflammatory changes were noted without significant additional collections. A chest radiograph showed no signs of consolidation or atelectasis, and venous Doppler ultrasound confirmed extensive septic thrombosis with compromised blood flow (Fig. 2).

### Surgical interventions

The first surgical procedure took place when the pediatric surgical team performed open drainage of the purulent collections in the neck and mediastinum. Multiple abscesses were drained during the intervention. However, postoperative recovery was slow, with an increase in the size of the mediastinal collections.

Due to this clinical deterioration, a second procedure was performed on, this time under general anesthesia, to drain the retropharyngeal abscess. During the intervention, carried out by the otorhinolaryngology team, 4 cc of organized fibrinoid secretion was drained. Despite the surgical interventions, the patient's recovery was slow, requiring adjustments in antimicrobial management.

### Medical treatment

The patient was treated with a broad-spectrum antibiotic regimen, including the administration of vancomycin for a period of 4 to 6 weeks. Additionally, clindamycin was administered intravenously, reaching day 24 of treatment. A 14-day course of cefepime was also completed. Antimicrobial coverage was adjusted based on cultures obtained from the abscess and blood cultures.

For the management of the jugular thrombosis, subcutaneous enoxaparin was administered, adjusted according to anti-Xa levels, which were initially subtherapeutic. The dose was 6.5 mg every 12 hours, with regular monitoring of anticoagulation levels.

Ventilatory support included the use of high-flow nasal cannula, which was temporarily suspended and restarted depending on the evolution of the respiratory pattern. To manage laryngeal stridor and airway inflammation, inhaled steroids were administered. Additionally, the patient received enteral nutrition via a nasogastric tube, with 90 cc administered every 3 hours. To control withdrawal syndrome and ensure comfort, a combination of dexmedetomidine, clonazepam, and levomepromazine was used.

### Complications

During his stay in the PICU, the patient suffered multiple complications. Septic shock, unresponsive to crystalloids, required intensive management with broad-spectrum antibiotics. He also experienced an episode of adrenal insufficiency, with a documented cortisol level of 0.6, which was successfully treated. Another issue identified was hypogammaglobulinemia, with low levels of IgG, without a family history of immunodeficiencies. Additionally, he developed a urinary tract infection caused by Klebsiella oxytoca with a natural phenotype, which was effectively treated.

The patient was discharged 1 month after admission, and the outpatient consultation showed no additional complications.

This case highlights the complexity of Lemierre syndrome in the pediatric population and the importance of early multidisciplinary management to improve recovery outcomes.

Differential diagnosis of Lemierre's syndrome through imaging, it's crucial to distinguish it from other conditions that may present similar radiological findings:

Deep neck space infections (Peritonsillar or retropharyngeal abscess):
Imaging: Contrast-enhanced CT shows a fluid collection with rim enhancement and potential air-fluid levels.
Differentiation: Unlike Lemierre's, there's often no internal jugular vein (IJV) thrombosis or septic emboli.
Nonseptic Internal Jugular Vein Thrombosis:
Imaging: Doppler ultrasound or CT shows a nonenhancing thrombus within the IJV.
Differentiation: Absence of systemic sepsis or distant septic emboli seen in Lemierre's.
Suppurative lymphadenitis:
Imaging: Enlarged, inflamed lymph nodes with central necrosis and peripheral enhancement on contrast CT.
Differentiation: No involvement of the IJV or pulmonary septic emboli.
Necrotizing Pneumonia or Lung Abscess:
Imaging: Chest CT reveals cavitary lesions with air-fluid levels, similar to Lemierre's septic emboli.
Differentiation: No associated IJV thrombosis or pharyngeal infection history.
Mediastinitis:
Imaging: CT shows soft tissue swelling, fluid collections, and gas tracking from the neck into the mediastinum.
Differentiation: Lemierre's rarely shows mediastinal extension without severe deep neck infection.
Endocarditis with Septic Pulmonary Emboli:
Imaging: Echocardiogram reveals valvular vegetations, and CT shows pulmonary nodules or cavitations.
Differentiation: Primary cardiac origin rather than oropharyngeal infection with IJV involvement.
Mononucleosis with tonsillar hypertrophy:
Imaging: Bilateral tonsillar enlargement and cervical lymphadenopathy on CT.
Differentiation: No IJV thrombosis or pulmonary embolic disease.
Kawasaki disease with cervical adenitis:
Imaging: Unilateral lymph node enlargement without IJV involvement or lung findings.
Differentiation: Absence of thrombophlebitis or septic emboli.
Grisel's syndrome (Atlantoaxial Subluxation):
Imaging: Cervical spine radiographs or CT show abnormal atlantoaxial alignment.
Differentiation: No pharyngeal infection, IJV thrombosis, or pulmonary involvement.
Key Imaging Techniques for Diagnosis:
Doppler ultrasound: First-line for detecting IJV thrombosis.
Contrast-enhanced CT (Neck and Chest): Gold standard for identifying IJV thrombosis, peritonsillar infection, and septic pulmonary emboli.
MRI: Useful for soft tissue assessment and venous thrombosis when radiation exposure is a concern.

## Discussion and conclusions

In the most recent cohort studies, it has been observed that *Fusobacterium necrophorum* is the causal pathogen in more than 80% of reported cases, highlighting the importance of early detection due to its high morbidity and mortality. Additionally, other pathogens, such as Streptococcus spp. and Staphylococcus aureus, have also been implicated, especially in cases associated with retropharyngeal abscesses or mediastinitis [4]. This is particularly relevant in pediatric patients, where the diagnosis of this disease may be delayed due to the similarity of symptoms with other more common infections, such as streptococcal pharyngitis or viral infections [5].

The use of advanced radiological imaging has revolutionized the management of Lemierre syndrome, allowing earlier identification of thrombosis and its complications, such as abscesses and septic spread. Contrast-enhanced computed tomography (CT) has proven to be a key tool in detecting venous thrombosis and deep abscesses in the neck and mediastinum [6]. In a case- control study conducted by Dool et al. [7], CT was able to identify the extent of thrombosis and guide surgical treatment in 90% of cases.

At the pediatric level, diagnosing Lemierre syndrome can be even more complex. In a retrospective analysis of a series of pediatric cases, it was observed that patients exhibited a faster clinical progression and a higher tendency to develop complications, such as mediastinal abscesses and pulmonary sepsis, compared to adults [8]. This pattern of more aggressive presentation underscores the importance of a multidisciplinary approach and the need for timely imaging studies to prevent clinical deterioration.

Moreover, managing Lemierre syndrome in the pediatric population requires a comprehensive surgical and medical approach. The use of broad-spectrum antibiotics that cover both anaerobic and aerobic bacteria has been recommended, along with regular imaging assessments to monitor disease progression [9]. In cases where venous thrombosis is extensive, the administration of anticoagulants has been suggested, though its use remains controversial, particularly in young children, due to the risk of bleeding (10).

This particular clinical case highlights the importance of rapid intervention in the context of a retropharyngeal abscess that progressed to complicated Lemierre syndrome, emphasizing the need to combine advanced diagnostic techniques with appropriate antibiotic management to improve the prognosis in pediatric patients.

Although rare, Lemierre syndrome represents a potentially life-threatening complication of oropharyngeal infections and requires a high index of suspicion for timely diagnosis and management. Imaging plays a pivotal role in early recognition and management of this condition. This case of a 2-month-old infant with Lemierre syndrome presents several notable aspects that warrant discussion:

Atypical presentation in infants: Lemierre syndrome is rare in such young patients. Most reported cases in the literature affect adolescents and young adults (Riordan, 2007). The presentation in this infant, with rapid progression from an initial bronchiolitis to a severe condition with a retropharyngeal abscess and jugular vein thrombosis, underscores the need to consider this entity even in very young patients with upper respiratory tract infections that evolve unfavorably. Imaging findings play a critical role in distinguishing Lemierre syndrome from other causes of neck infections.

Radiological diagnostic challenges: The case highlights the importance of advanced imaging techniques in diagnosis. Contrast-enhanced MRI was crucial in identifying the extent of the retropharyngeal abscess and the presence of internal jugular vein thrombosis, findings characteristic of Lemierre syndrome. MRI provided additional soft tissue resolution, aiding in the assessment of associated inflammatory changes and possible complications. These imaging modalities, as noted by Dool et al. [10], are essential for timely and accurate diagnosis.

Role of interventional radiology: The involvement of interventional radiology was key in both diagnostic and therapeutic aspects. Ultrasound-guided vascular access and possible drainage procedures highlight the importance of radiological expertise in managing complex cases of Lemierre syndrome.

Multidisciplinary management: The approach to this case involved multiple specialties, including pediatric intensive care, pediatric surgery, otolaryngology, and interventional radiology. This collaboration was crucial for comprehensive management of the patient, underscoring the need for a multidisciplinary approach in complex cases of Lemierre syndrome, as suggested by Karkos et al. [11].

Antibiotic strategy: The use of broad-spectrum antibiotics, including vancomycin and clindamycin, aligns with current recommendations for the treatment of Lemierre syndrome, considering the frequent etiology of Fusobacterium necrophorum and other anaerobes [12].

Anticoagulation: The decision to anticoagulate the patient with enoxaparin reflects the ongoing debate regarding the use of anticoagulants in Lemierre syndrome. Although controversial, especially in pediatric patients, some studies suggest benefits in cases of extensive thrombosis [13].

Complications and follow-up: Additional complications such as adrenal insufficiency and hypogammaglobulinemia highlight the need for close and prolonged follow-up in these patients. The possibility of long-term sequelae, including immunological deficiencies, warrants continuous evaluation.

Implications for radiological practice: This case highlights the importance of maintaining a high index of suspicion for Lemierre syndrome in pediatric patients with upper respiratory tract infections that do not respond to conventional treatment.

Radiologists must be vigilant in recognizing the hallmark findings of retropharyngeal abscess and internal jugular vein thrombosis on imaging, enabling early and aggressive intervention when this condition is suspected.

In conclusion, this case of Lemierre syndrome in an infant highlights the atypical presentation of this “forgotten disease” in very young patients, the associated diagnostic and therapeutic challenges, and the importance of a multidisciplinary approach. Future studies could explore the possible relationship between viral infections such as SARS-CoV-2 and the development of Lemierre syndrome, as well as optimizing imaging and management strategies in the pediatric population.

## Patient consent

We declare that we have obtained the respective informed consents regarding clinical history and images, endorsed by the hospital's ethics committee.
